# An Existential-Humanistic View of Personality Change: Co-Occurring Changes with Psychological Well-Being in a 10 Year Cohort Study

**DOI:** 10.1007/s11205-014-0648-0

**Published:** 2014-05-16

**Authors:** Hilda Osafo Hounkpatin, Alex M. Wood, Christopher J. Boyce, Graham Dunn

**Affiliations:** 1 Centre for Biostatistics, Institute of Population Health, University of Manchester, Jean McFarlane Building (1st Floor), Oxford Road, Manchester, M13 9PL UK; 2 Behavioural Science Centre, Stirling Management School, University of Stirling, Stirling, FK9 4LA Scotland, UK

**Keywords:** Personality change, Psychological well-being scale, Well-being, Big Five, Existential, Humanistic

## Abstract

Increasingly, psychological research has indicated that an individual’s personality changes across the lifespan. We aim to better understand personality change by examining if personality change is linked to striving towards fulfilment, as suggested by existential–humanistic theories of personality dynamics. Using the Wisconsin Longitudinal Study, a cohort of 4,733 mid-life individuals across 10 years, we show that personality change was significantly associated with change in existential well-being, represented by psychological well-being (PWB). Moreover, personality change was more strongly related to change in PWB than changes in other well-being indicators such as depression, hostility and life satisfaction. Personality changed to a similar degree and explained greater variation in our well-being measures than changes in socioeconomic variables. The findings indicate personality change is necessary for the holistic development of an individual, supporting a greater need to understand personality change and increasing room for use of personality measures as indicators of well-being and policy making.

## Introduction

Within psychology, the view of personality as stable throughout life is rapidly changing to one where traits react fluidly to life circumstances (Caspi 1998; Caspi and Bem 1990; Roberts et al. 2008). Despite a mass of evidence that suggests an individual’s personality changes across the complete lifespan (Lucas and Donnellan 2011; Roberts et al. 2006; Specht et al. 2011), core personality traits are still generally considered ‘relatively enduring’, particularly in disciplines outside psychology. As a result of this, the use of personality change measures in well-being research has been limited, with most studies utilising personality measures at one time point to predict well-being outcomes (Boyce and Wood 2011; deBeurs et al. 2005; DeNeve and Cooper 1998; Friedman et al. 2010; Steel et al. 2008) and only a few studies exploring the effect of personality change on well-being.

Studies that have investigated personality change have found an association with subjective well-being measures such as life satisfaction (Boyce et al. 2013; Heller et al. 2007; Specht et al. 2013; vanAken et al. 2006), self-rated health (Berg and Johansson 2014; Magee et al. 2013; Turiano et al. 2012), self-efficacy (Hutteman et al. 2014), psychological turning points (Allemand et al. 2010; Sutin et al. 2010) as well as physical and mental health outcomes (Human et al. 2013; Mroczek and Spiro 2007). Evidence for an association between well-being and personality has been taken to support the social investment perspective on personality development (Roberts and Wood 2006; Roberts et al. 2005) which suggests that committing and successfully adapting to social roles such as marriage and work drives personality development. Whilst the social investment theory considers the effect of societal-determined expectations and goals on personality change, it does not address the importance of striving for authentic, self-concordant goals for personality change. Such a relation forms the basis of an alternative explanation for personality change, proposed by existential and humanistic theories which have not previously been introduced into the contemporary empirical literature on the malleability of personality.

Taken together, the existential and humanistic theories propose that each individual has the freedom and responsibility to transcend the meaninglessness of their existence. Personality change is thought to occur when the individual confronts meaningless in life and has to decide for themselves how to shape their life.^1^ If the individual chooses to strive towards fulfilment, personality is likely to develop in potentially positive ways (i.e., perhaps becoming more open to opportunities or more extraverted) because the individual recognises their capacity to choose their own future and is able to take full advantage of opportunities to find meaning to their existence. Alternatively, if the individual is consumed with feelings of despair and fails to engage with themselves and the world around them to achieve their full potential, this may result in changes in the opposite direction (i.e., becoming less open and more introverted). Associating personality change with changes in such ways of functioning would be part of a theoretical movement from seeing personality change as a biological maturation or social investment process towards seeing such change as part of a holistic development of the person in ways that are right for the individual (existential well-being) (Deci and Ryan 1985, 2000; Joseph and Linley 2005).

The existential-humanistic theory of personality change can be tested using a measure of psychological well-being (PWB). Waterman (1984, 1993) defines PWB as concerned with the feelings associated with an individual’s strive to grow and fully develop oneself amid life challenges. PWB encompasses an individual’s perception of engagement with the self, environment and others (Keyes et al. 2002; Ryan and Deci 2001), thus capturing existential well-being. In terms of measurement, Ryff (1989), Ryff and Keyes (1995) operationalize PWB as comprising *autonomy* (the extent to which one is self-determining and independent), *environmental mastery* (competence in managing the environment and presented opportunities), *personal growth* (possessing feelings of continued developments), *positive relations* (having strong social ties), *purpose in life* (having goals in life or a sense of directedness) and *self*-*acceptance* (possessing a positive attitude toward the self) (Ryff and Keyes 1995).

In this paper we report on a study that seeks to better understand the relationship between personality change and well-being change through linking changes in personality to an individual’s existential engagement with the world, as represented by changes in PWB. We additionally aim to assess the use of personality change measures as well-being indicators and targets for intervention, through (a) quantifying the size of personality change relative to socioeconomic metrics commonly used in well-being research and (b) comparing the predictive value of changes in personality and socioeconomic factors on changes in PWB. In order to quantify an effect size as large or small, direct comparisons with effect sizes of other variables of interest must be made (Cohen 1992; Glass et al. 1981). Recently, Boyce et al. (2013) have been the first to compare the magnitude of personality change with that of socioeconomic indicators that are widely considered changeable (e.g. income, marital employment status). They find that personality changes at least as much as socioeconomic factors across a wide age range. Here, we specifically examine whether personality changes more than socioeconomic factors during midlife. Furthermore, we examine how personality change relates to changes in other well-being measures such as depression, hostility, and life satisfaction in order to assess the importance of personality change for PWB over other well-being measures.

## Materials and Methods

### Participants and Procedure

Participants were from the Wisconsin Longitudinal Study (WLS), a cohort of 10,317 individuals who graduated from Wisconsin high schools in 1957 (Little 1958; Sewell and Orenstein 1965). The sample is representative of white Americans living in Wisconsin who were born in 1938–1940 and completed at least 12 years of schooling. The WLS contains both measures of personality and socioeconomic data, allowing us to make direct comparisons of personality change and socioeconomic change in a large sample. Personality measures were collected during the 1992 and 2004 time waves. Therefore, our analyses only used data from these two waves. Participants who gave responses for all variables of principal interest at both time points (*N* = 4,733) were analysed for this study. This sample consisted of a similar proportion of males and females as in the main sample. Participants were approximately 53–54 and 64–65 years old in 1992 (Time 1) and 2004 (Time 2) respectively.

### Measures

#### Big Five personality traits

Personality traits were assessed based on the Big Five Inventory (BFI; John et al. 1991). Respondents were asked 29 questions on the five traits—namely neuroticism, extraversion, openness, agreeableness and conscientiousness—for which responses ranged from noe ‘strongly agree’ to 6 ‘strongly disagree’. The neuroticism subscale consisted of 5 questions, and subscales for the remaining traits comprised 6 questions each. Examples of the questions asked are as follows: *neuroticism* (e.g., “do you agree that you see yourself as someone who is emotionally stable, not easily upset”), *extraversion* (e.g., “do you agree that you see yourself as someone who is talkative”), *openness* (e.g., “do you agree that you see yourself as someone who has an active imagination”), *agreeableness* (e.g., “do you agree that you see yourself as someone who is generally trusting”), *conscientiousness* (e.g., “do you agree that you see yourself as someone who does a thorough job”). Scores were summed for individuals who responded to at least one of the questions for each trait and then averaged by the number of questions answered. The BFI allows the five dimensions of personality to be measured efficiently and flexibly when there is no need for a more differentiated measurement of individual facets (John et al. 1991; John et al. 2010; John and Srivastava 1999). The BFI is used widely in research settings and has been shown to be reliable, easier to understand and shorter than other Big Five scales (Benet-Martinez and John 1998; Soto et al. 2008).

#### *Psychological Well*-*Being*

Psychological well-being (PWB) was assessed through a 42-item version of Ryff’s PWB scales (Ryff and Keyes 1995). For our analyses, we focused only on the questions which were asked at both time points (as in the study by Springer et al. 2011, so that change in scores across the two time points could not be attributed to a difference in wording of the questions. A total of 19 questions were asked at both time points (4 questions for the *purpose in life* subscale and 3 questions for the remaining subscales. Sample items for each subscale were as follows: *autonomy* (e.g., “do you agree that you have confidence in your decisions even if contrary to general consensus?”), *environmental mastery* (e.g., “do you agree that you have been able to create a lifestyle that is much to your liking?”), *personal growth* (e.g., “do you agree that you have the sense that you have developed a lot as a person over time), *positive relationships* with others (e.g., “do you agree that you enjoy personal and mutual conversations with family and friends?”), *purpose in life* (e.g., “do you agree that you sometimes feel as if you’ve done all there is to do in life?”) and *self*-*acceptance* (e.g., “do you agree that, in general, you feel confident and positive about yourself?”). Responses ranged from 1 to 6, higher scores indicating higher well-being. An average score was calculated for each subscale at each time point if at least one of the questions were answered. Internal consistency was acceptable, with alpha coefficients as follows: autonomy (α = .60), environmental mastery (α = .65), personal growth (α = .64), positive relations (α = .65), purpose in life (α = .68), self-acceptance (α = .68), suggesting reliability of the instrument as a measure of PWB. Alpha coefficients are slightly lower than may be achieved using the full version of the scale, though the abbreviated scales have been shown to have a high correlation with the original scale (Ryff and Keyes 1995).

#### Life Satisfaction

A single item—“do you agree that when you look at the story of your life, you are pleased with how things have turned out?”—was used, to which responses ranged from 1 to 6, a score of 1 corresponding to highest satisfaction. These scores were reversely coded for our analysis so that highest score would correspond to highest satisfaction. This measure is particularly useful as it requires participants to consider their satisfaction over their entire life course, thereby producing stable estimates. Although single item measures are less stable than multi-item scales, Lucas and Donnellan (2012) have estimated the reliability of single item life satisfaction measures from four large scale nationally representative longitudinal studies and have on average found estimates of .72, which exceeds the cut-off value of .70 as an acceptable reliability for measures with moderate levels of reliability (Lance et al. 2006). Furthermore, research indicates single item measures correlate highly with multi-item life satisfaction measures (van Beuningen 2012) and other indicators of well-being (Diener et al. 2009).

#### Depression

A 20-item version of the Centre for Epidemiological Studies-Depression (CES-D) was used. Respondents were asked how frequently they experienced depressive symptoms (sample item: “how many days this week did you feel lonely”). Scores were reverse coded as appropriate. Scores were summed for subjects who responded to at least 3 items and then averaged by the number of items answered. CES-D is a highly reliable measure, having 100 % sensitivity and 88 % specificity in detecting clinical depression as assessed by nurse-clinicians (McDowell and Kristjansson 1996; Radloff 1977). Alpha reliability for our measure of depression was α = .87 and α = .86 at Time 1 and Time 2 respectively.

#### Hostility

Hostility was assessed using a 3 item scale: “how many days during the past week did you feel irritable or likely to argue”, “how many days during the past week did you feel like telling someone off?”, and “how many days during the past week did you feel angry or hostile for several hours at a time?” Scores from the three items were summed and averaged for each individual. This scale gives a reliable measure of hostility, with alpha coefficient of .78.

#### Socioeconomic Variables

Total annual household income was log-transformed prior to analyses. Household size and socioeconomic data such as current employment status (employed or unemployed), level of educational achievement (high school, <1 year college, college without bachelor degree, bachelor degree, graduate degree or above), marital status (married, separated, divorced, widowed, never married) and retirement (partly retired, completely retired, not retired at all) was controlled for in our analyses. Gender was accounted for as having a non-changing effect. Physical health (based on whether participants were ever diagnosed by a medical doctor as having long-standing illness such as cancer, chronic liver trouble, chronic heart trouble, anaemia, asthma, arthritis, diabetes, bronchitis/emphysema, circulation problems, back trouble, ulcers, allergies, kidney or bladder problems, colitis, high blood pressure and multiple sclerosis) was also controlled for in our analyses, as health status would be expected to affect both personality and well-being. Participants who did not provide physical health data at both time points (*N* = 206) were excluded from our regression analyses.^2^

### Statistical Procedure

Statistical analyses were conducted using STATA v.11 (StataCorp 2009). Stability for each of the Big Five traits was estimated using the Pearson correlation between scores at the two time points. This panel consisted of a total of 9,466 observations, corresponding to 4,733 individuals. We created dummy variables for all categorical predictors (e.g., 5 dummy variables—‘married’, ‘separated’, ‘divorced’, ‘widowed’ and ‘never married’ were generated for marital status). To determine whether personality traits can be considered as time-varying for statistical analyses purposes, we assessed the extent to which personality variables varied between compared to within individuals by dividing the standard deviation of the personality variable between individuals by the standard deviation within the individual. A low between-to-within ratio suggests that a variable changes more within an individual than between individuals over time and therefore can be incorporated into analyses that focus on within-individual change (Boyce 2010; Boyce et al. 2013; Plumper and Troeger 2007). A large between-to-within ratio indicates a time invariant variable.

We further compare our stability ratios for the personality variables with that of socioeconomic indicators which are generally considered malleable, such as household income and employment status. Table 1 presents a summary of our stability ratios for the well-being, personality and socioeconomic variables across the sample.

**Table 1:** Summary statistics across sample measured at two time points

Variable	Overall μ	Overall σ	Between σ	Within σ	‘Between to within variation’ ratio
Log-transformed household income	10.32	2.56	2.08	1.50	1.39
Household income ($)	69,778	71,413	61,252	36,720	1.67
Unemployment	0.28	0.45	0.27	0.36	0.73
Neuroticism	3.07	0.95	0.86	0.39	2.21
Extroversion	3.82	0.88	0.83	0.31	2.68
Openness	3.63	0.79	0.73	0.30	2.43
Agreeableness	4.77	0.72	0.65	0.31	2.10
Conscientiousness	4.85	0.68	0.61	0.29	2.10
Married	0.82	0.39	0.35	0.15	2.31
Separated	0.00	0.06	0.04	0.04	1.06
Divorced	0.10	0.30	0.28	0.10	2.66
Widowed	0.05	0.21	0.17	0.12	1.44
Never married	0.04	0.20	0.20	0.02	11.56
Partly retired	0.11	0.31	0.21	0.23	0.93
Completely retired	0.25	0.43	0.25	0.35	0.72
Not retired	0.64	0.48	0.26	0.40	0.64

Unstandardised score presented. *μ* = mean, *σ* = standard deviation. *N* = 9,466

Next we examined whether changes in an individual’s personality, income, education, marital and physical health status was associated with change in their well-being. Personality and well-being scores were standardised across the entire population (to have a mean of 0 and standard deviation of 1) to facilitate interpretation of results. Difference scores were generated for each socioeconomic, well-being and personality variables, which represented the change in measure in that variable between Time 2 and Time 1 for each individual. For categorical variables, the difference in dummy variables was used such that an individual who was married at Time 1 but separated at Time 2 would have a value of ‘−1’ for the ‘married’ dummy variable, ‘1’ for the ‘separated’ dummy variable and ‘0’ for the remaining categories. Difference scores is the most efficient way to deal with unobserved confounders when using two panel data (Angrist and Pischke 2008; Rogosa and Willet 1983; Wooldridge 2003).

Tables 2 and 3 present the results of our difference scores analysis. Specifically, we fit three models for each well-being measure; Model 1 estimates the association between changes in socioeconomic variables (i.e. changes in log-transformed income, unemployment, education, marital, retirement, physical health status) and change in the specified well-being variable. Model two estimates the association between change in the well-being variable and changes in the personality variables. Model three estimates the association between change in the specified well-being variable and changes in the personality variables, additionally adjusting for changes in the socioeconomic variables.

**Table 2:** Estimation of PWB change on changes in socioeconomic and personality variables

Predictor variables	Outcome variables								
	Autonomy			Environmental mastery			Personal growth		
	Model 1	Model 2	Model 3	Model 1	Model 2	Model 3	Model 1	Model 2	Model 3
	(1a)	(2a)	(3a)	(1b)	(2b)	(3b)	(1c)	(2c)	(3c)
Controls	Yes	No	Yes	Yes	No	Yes	Yes	No	Yes
Log-transformed income	0.01 (0.01)		0.01 (0.01)	0.01 (0.01)		0.01 (0.00)	0.01 (0.01)		0.01 (0.00)
Unemployed	0.11 (0.06)		0.10 (0.06)	0.09 (0.06)		0.06 (0.06)	0.04 (0.06)		0.02 (0.06)
Neuroticism		−0.07* (0.02)	−0.07* (0.02)		−0.15** (0.02)	−0.14** (0.02)		−0.05* (0.02)	−0.05* (0.02)
Extroversion		0.09** (0.02)	0.09** (0.02)		0.12** (0.02)	0.12** (0.02)		0.11** (0.02)	0.11** (0.02)
Openness		0.06* (0.02)	0.05* (0.02)		0.03 (0.02)	0.03 (0.02)		0.08** (0.02)	0.08** (0.02)
Agreeableness		0.02 (0.02)	0.02 (0.02)		0.08** (0.02)	0.08** (0.02)		0.12** (0.02)	0.12* (0.02)
Conscientiousness		0.09** (0.02)	0.09* (0.02)		0.14** (0.02)	0.14** (0.02)		0.10** (0.02)	0.09** (0.02)
Constant	0.06* (0.03)	0.04* (0.01)	0.05* (0.03)	0.04 (0.03)	0.02 (0.01)	0.04 (0.03)	0.05 (0.03)	0.01 (0.01)	0.05 (0.02)
Individuals	4504	4504	4504	4504	4504	4504	4492	4492	4492
R-squared	0.01	0.03	0.04	0.01	0.07	0.08	0.01	0.05	0.06

Predictor variables	Outcome variables								
	Positive relations			Purpose in life			Self-acceptance		
	Model 1	Model 2	Model 3	Model 1	Model 2	Model 3	Model 1	Model 2	Model 3
	(1d)	(2d)	(3d)	(1e)	(2e)	(3e)	(1f)	(2f)	(3f)
Controls	Yes	No	Yes	Yes	No	Yes	Yes	No	Yes
Log-transformed income	0.01 (0.00)		0.01 (0.00)	0.00 (0.01)		0.00 (0.01)	−0.00 (0.01)		−0.00 (0.00)
Unemployed	0.09 (0.05)		0.06 (0.05)	0.00 (0.06)		−0.02 (0.06)	0.05 (0.07)		0.03 (0.06)
Neuroticism		−0.10** (0.02)	−0.10** (0.02)		−0.08** (0.02)	−0.08** (0.02)		−0.11** (0.02)	−0.11** (0.02)
Extroversion		0.14** (0.02)	0.14** (0.02)		0.12** (0.02)	0.12** (0.02)		0.12** (0.02)	0.11** (0.02)
Openness		0.03 (0.02)	0.03 (0.02)		0.08** (0.02)	0.07** (0.02)		0.05* (0.02)	0.05* (0.02)
Agreeableness		0.15** (0.02)	0.15** (0.02)		0.11** (0.02)	0.11** (0.02)		0.11** (0.02)	0.11** (0.02)
Conscientiousness		0.05* (0.02)	0.05* (0.02)		0.11** (0.02)	0.11** (0.02)		0.10** (0.02)	0.10** (0.02)
Constant	−0.02 (0.02)	0.01 (0.01)	−0.02 (0.02)	0.07* (0.03)	0.03* (0.01)	0.07* (0.02)	0.01 (0.03)	0.01 (0.01)	0.01 (0.03)
Individuals	4505	4505	4505	4495	4495	4495	4494	4494	4494
R-squared	0.01	0.07	0.08	0.01	0.07	0.07	0.01	0.06	0.07

Standardised estimates (SE) * significant at 5 % ** significant at 1 %. Model 1: Socioeconomic model, additionally adjusting for education, household size, marital status, retirement status and physical health variables (not shown). Model 2: Personality model, *not* adjusting for education, household size, marital status, retirement status and physical health variables. Model 3: Joint regression for personality and socioeconomic variables, additionally adjusted for education, household size, marital status, retirement status and physical health variables (not shown)

**Table 3:** Estimation of changes in life satisfaction, depression, hostility on changes in socioeconomic and personality variables

Predictor variables	Outcome variables								
	Life satisfaction			Depression			Hostility		
	Model 1	Model 2	Model 3	Model 1	Model 2	Model 3	Model 1	Model 2	Model 3
	(1g)	(2g)	(3g)	(1h)	(2h)	(3h)	(1i)	(2i)	(3i)
Controls	Yes	No	Yes	Yes	No	Yes	Yes	No	Yes
Log-transformed income	0.00 (0.01)		0.00 (0.01)	−0.01 (0.01)		−0.01 (0.01)	−0.00 (0.01)		−0.00 (0.01)
Unemployed	−0.04 (0.07)		−0.05 (0.07)	−0.05 (0.07)		−0.02 (0.06)	0.01 (0.07)		0.02 (0.06)
Neuroticism		−0.06* (0.02)	−0.06* (0.02)		0.22** (0.02)	0.22** (0.02)		0.14** (0.02)	0.13** (0.02)
Extroversion		0.06* (0.03)	0.06* (0.03)		−0.09** (0.02)	−0.09** (0.02)		−0.05 (0.02)	−0.04 (0.02)
Openness		0.01 (0.02)	0.02 (0.02)		−0.01 (0.02)	−0.01 (0.02)		−0.09* (0.03)	−0.09* (0.03)
Agreeableness		0.05* (0.02)	0.05* (0.02)		−0.02 (0.02)	−0.03 (0.02)		−0.11** (0.03)	−0.11** (0.03)
Conscientiousness		0.05* (0.02)	0.05* (0.02)		−0.08** (0.02)	−0.07** (0.02)		0.00 (0.02)	0.00 (0.02)
Constant	0.02 (0.03)	−0.01 (0.02)	0.02 (0.03)	−0.02 (0.03)	0.00 (0.01)	−0.02 (0.03)	0.04 (0.03)	0.00 (0.02)	0.04 (0.03)
Individuals	3511	3511	3511	4449	4449	4449	4406	4406	4406
R-squared	0.01	0.01	0.03	0.03	0.06	0.08	0.01	0.03	0.04

Standardised estimates (SE) * significant at 5 % ** significant at 1 %. Model 1: Socioeconomic model additionally adjusted for education, household size, marital status, retirement status and physical health variables (not shown). Model 2: Personality model, *not* adjusting for education, household size, marital status, retirement status and physical health variables. Model 3: Joint regression for personality and socioeconomic variables, additionally adjusted for education, household size, marital status, retirement status and physical health variables (not shown)

## Results

Stability of personality scores across time were as follows: 0.68 for neuroticism, 0.74 for extraversion, 0.71 for openness, 0.61 for agreeableness, 0.62 for conscientiousness. These coefficients were comparable to those found in similar study by Roberts and delVecchio (2000). Our stability coefficients represent the correlation between the mean personality score at Time 1 and Time 2 and therefore indicate that personality measures across the sample are generally stable over time. However, the high stability coefficients do not preclude the possibility of personality changes within an individual (Ozer 1986), which is the focus of our study. The less than perfect (i.e. *r* < 1.0) stability across the two time points further suggest there may be are individual-level changes in personality. In order to explore this, we examined the number of individuals who showed reliable change (i.e. true change not due to measurement error) in personality measures from Time 1 to Time 2 and the magnitude of this change. Table 4 illustrates the percentage of individuals who experienced true change in personality measures and the lowest and highest magnitude of change (in standard deviation) for these individuals. Our results in Table 4 shows that a proportion of the sample experience change in personality of considerable magnitude. Through the ‘between-to-within variation ratio’ in Table 1, we also show that even at midlife, personality changes as much as other indicators that have traditionally been used to predict human outcomes. Our between-to-within stability ratios were lower for personality traits than for the different categories of educational achievement and marital status in our sample, indicating that an individual’s personality is more likely to change from Time 1 to Time 2 than their educational achievement or marital status.

**Table 4:** Individual differences in personality traits

	Decreased	Increased	No change	Magnitude of change in standard deviation	
				Min	Max
Neuroticism	16.7	7.3	76.0	1.0	3.8
Extraversion	8.4	6.3	85.3	1.0	3.2
Openness	4.6	3.0	92.4	1.0	4.0
Agreeableness	10.0	11.1	78.9	0.8	4.7
Conscientiousness	11.5	6.9	81.6	0.8	4.7

*N* = 4,733. Percentages of individuals who decreased, increased, or showed no reliable change in personality and the magnitude of this change

Table 2 demonstrates that personality change relates to significant changes in well-being. Models 2a–2f in Table 2 show that personality alone explained 3–7 times more variation (indicated by R-squared values) in the PWB subscales in our sample than socioeconomic and health indicators together (Models 1a–1f). For example, in Model 2a in Table 2, personality change explained 3 % of the variation in an individual’s level of autonomy, while changes in socioeconomic variables (Model 1a in Table 3) explained only 1 % of the within-person variation. Similarly, personality change explained 7 % of the within-person variation in environmental mastery (Model 2b), 5 % of the within-person variation in personal growth (Model 2c), positive relations (Model 2d) and purpose in life (Model 2e), and 6 % of the within-person variation in self-acceptance (Model 2f) over the 10 years period. Socioeconomic variables together explained only 1 % of the within-person variation in each of the PWB subscales (Models 1a, 1c–1f). Our R squared values are similar to those found in similar models which estimate variation within individuals (Boyce et al. 2013; Ferrer-i-Carbonell and Frijters 2004) and are used here to highlight the stronger relationship between well-being change and change in personality measures compared to change in socioeconomic factors. Furthermore, Models 1a–1f in Table 2 indicates that change in log-transformed income and unemployment status were not significant predictors of change in any of the PWB subscales while Models 2a–2f in Table 2 shows that personality change was significantly associated with change in each of the PWB subscales. For example, in the case of purpose in life (Model 2e), a one standard deviation increase in extroversion, openness, agreeableness and conscientiousness was significantly associated with a 0.12, 0.08, 0.11 and 0.11 standard deviation increase in purpose in life respectively, and a one unit increase in neuroticism was significantly associated with a 0.08 standard deviation decrease in purpose in life. In Models 3a–3f, we further show that personality change remained significantly associated with changes in all PWB scales (effect sizes ranging from 0.04 to 0.15 standard deviations) even after controlling for changes in socioeconomic variables, whereas changes in socioeconomic indicators were only significantly associated with changes in positive relations (becoming partly retired being associated with a 0.08 standard deviation increase in positive relations compared to not being retired).

The importance of personality change for PWB is highlighted by examining how personality change relates to other well-being measures (life satisfaction, depression, hostility) compared to PWB. Models 2g–2i in Table 3 shows that personality change explained only as much within-person variation in life satisfaction, twice as much variation in depression and three times as much variation in change in hostility than change in socioeconomic variables (Models 1g–1f).

## Discussion

This study extends earlier research suggesting that personality change is in fact meaningful. We use a midlife population, an age group for which research on personality development is limited. Midlife presents an important period for personality development (Lachman 2004; Neugarten 1968) as it is associated with many biological, physical, work, social and psychological changes, amongst others which in turn may result in changes in personality (see Roberts and delVecchio 2000 and Allemand et al. 2007 for a discussion on mechanisms of trait consistency and change in midlife) as well as a period where individuals seek a sense of identity as they reflect on their lives. Obtaining clarity of self and striving towards a fulfilled life is associated with favourable changes in personality, and may be protective of any negative changes associated with midlife. Therefore, studying whether and how personality changes across midlife can give insight into how individuals are coping with midlife challenges.

Through our stability ratios in Table 1, we illustrate that personality changes to a similar extent as socioeconomic variables during midlife. In our sample, personality variables changed more than marital and education status and almost as much as income across 10 years. We further show that personality change is not only an indicator of change in life satisfaction as previously shown, but associated with changes in a wider range of measures over time, specifically PWB, even after adjusting for socioeconomic variables. In our sample, personality change explained up to seven times as much within-person variation in PWB than socio-economic variables, 2–3 times as much within-person variation in depression and hostility and as much within-person variation in life satisfaction. The overall findings highlight the importance of personality change for PWB as well as the need to distinguish between the different well-being constructs (Kahneman and Deaton 2010). The results provide empirical support for existential-humanistic theories of personality change, indicating that personality change is essential to an individual’s strive towards a fulfilled life; an increase in neuroticism indicating poor existential engagement with the world and increases in the remaining traits suggesting positive existential well-being. For all well-being measures, personality change was a better indicator of well-being change than change in socioeconomic variables. Taken together, the results show that an individual’s personality changes over time and that these changes are strongly related to changes in the individual’s existential well-being. These findings emphasise the importance of personality in psychological functioning during midlife, reiterating the need to integrate personality measures into well-being research.

### Limitations

Our study examines the association between changes in personality and well-being using two time points. Therefore we do not model how these variables change continuously but rather across a 10 years period. However, this could be seen as an advantage as we study long term changes in personality rather than temporary changes due to life events. Secondly, the use of a single item measure is a limitation of the dataset. Single item measures of life satisfaction are considered less stable and correlate less strongly with socioeconomic variables such as income and education (Pinquart and Sorensen 2000) than multi-item measures. However, this dataset was chosen as it is a large sample which provides data on life satisfaction, PWB and personality at two time points, allowing us to examine the association between these measures across time. A third limitation is that personality measures may be influenced by mental health status (Fergusson et al. 1989; Hirschfeld et al. 1983)—a depressed individual may report higher scores for neuroticism than they would in their pre-morbid state, since the individual’s mental state may result in more neurotic perceptions of themselves than usual. This would mean that an apparent change in self-reports of personality traits could be due to the effect of a mental disorder rather than associated changes in environmental circumstances or existential struggles. However, Fergusson et al. (1989) show that even after correcting for the effect of current mental state on neuroticism, neuroticism still remained a significant predictor of depression. Fourth, we excluded a large proportion of the sample (54 %) from our analyses due to missing data. To explore if individuals included in our analyses were different to those excluded from the analyses, we regressed an inclusion variable (which indicates whether subjects are included in the analyses) on each of our well-being outcomes and all control variables. These regression models indicated that education, employment status and physical health were predictors of inclusion into the analyses. For each outcome variable, a weight was then generated from the inverse of the predicted probability of the model predicting inclusion into the analyses. These weights were then included in our difference score models to account for missing data. The ‘weighted’ models produced similar results to our complete case analyses (Tables 2, 3), except for the regression predicting change in depression, which indicated that becoming unemployed was associated with a 0.20 standard deviation decrease in depression status (*p* < 0.01), compared to a 0.06 standard deviation decrease in depression in the complete case analysis. However, despite the substantive difference in the regression coefficient for unemployment, the weighted model personality change explained the same amount of variation in depression status as in the complete case analysis. Finally, our analysis can not make any causal inferences or direction about the personality-well-being relationship; whereas we have discussed the life choices people make as antecedents of personality change, it may be that certain personality traits facilitate the growth and development process. More research would be needed to confirm the causal pathways between personality change and well-being. Furthermore, we note that associating PWB change and personality change is consistent with other theories of personality development. We merely present the existential-humanistic theory as an alternative explanation for personality development.

## Conclusion

Personality change has important implications for public policy making, particularly as they may provide intrinsic measures of how people are engaging with the world. While policy has focused on inevitable socioeconomic changes across time this research indicates that it is the concurrent personality changes that are more strongly related to well-being. Public health interventions aimed at targeting specific aspects of personality such as social support (Oddone et al. 2011), cognitive training (Jackson et al. 2012), self-regulation (Baumeister et al. 2006) may be the key to helping individuals better cope with changes in life circumstances and improve their well-being as they mature.

## Footnotes

1 See Wong (2006) for a discussion on how this perspective of personality change emerges from the work of Victor Frankl, Abraham Maslow, Rolo May, and Carl Rogers.

2 Participants who did not respond to health questions had a lower mean income level, lower personality and PWB scores and higher hostility and depression scores than those who did. To assess whether including these individuals would alter our analysis results, we first regressed the odds of responding to physical health questions on well-being, personality and socioeconomic measures across our sample. We then estimated the predicted probability of this regression repeated our difference score analysis, this time including a variable for the inverse of the predicted probability. Results were similar to our complete case analyses.
